# Canine brucellosis in three littermates, case report

**DOI:** 10.3389/fvets.2022.958390

**Published:** 2022-10-06

**Authors:** Lindsey T. Graham, Samantha N. Vitale, Kari D. Foss, Devon W. Hague, Kimberly M. Anderson, Carol W. Maddox

**Affiliations:** ^1^Department of Veterinary Clinical Medicine, College of Veterinary Medicine, University of Illinois Urbana-Champaign, Urbana, IL, United States; ^2^Department of Small Animal Clinical Sciences, College of Veterinary Medicine, University of Tennessee, Knoxville, TN, United States; ^3^Department of Pathobiology, College of Veterinary Medicine, University of Illinois Urbana-Champaign, Urbana, IL, United States

**Keywords:** *Brucella canis*, discospondylitis, marbofloxacin, rapid slide agglutination test, magnetic resonance imaging, case report

## Abstract

Three adult littermates were diagnosed with *Brucella canis, two* of which were diagnosed with discospondylitis. The first littermate, a 2-year-old spayed-female Labrador Retriever, was evaluated for progressive episodes of cervical pain, lethargy, reported circling to the right, and a right-sided head tilt. Magnetic resonance imaging (MRI) of the cervical spine revealed changes consistent with discospondylitis at C6-C7. MRI of the brain was unremarkable and cerebrospinal fluid analysis was declined. *Brucella* spp. was isolated from aerobic and Brucella blood cultures. PCR performed on the isolate identified *Brucella canis* and indirect fluorescent antibody (IFA) testing for *Brucella canis* also confirmed the species. Patient #1 was treated with doxycycline and marbofloxacin for 1 year. Clinical signs returned 2-years after diagnosis. Following the diagnosis of patient #1, a known littermate (patient #2) was tested for *Brucella canis*. Patient #2 was 2 years old and asymptomatic at the time of diagnosis. Aerobic and *Brucella* spp. cultures, PCR, and IFA were obtained and were diagnostic for *Brucella canis*. A 6-month course of marbofloxacin and doxycycline was implemented. The patient remained PCR positive following 4 months of treatment and repeat cultures were planned following 6 months of treatment; however, the patient was lost to follow-up. A third littermate (patient #3) was identified by the family of patient #1. Patient #3 was evaluated at 18 months of age for a 6-month history of progressive lumbosacral pain. Spinal radiographs revealed discospondylitis of the C3-C4, T12-T13, and L7-S1 vertebral endplates. Computed tomography (CT) of the lumbosacral spine was also consistent with discospondylitis at L7-S1. *Brucella canis* serologic testing consisting of rapid slide agglutination test, 2ME-rapid slide agglutination test, and cytoplasmic agar gel immunodiffusion was positive. Enrofloxacin was administered for 7 months and was discontinued thereafter based on radiographic evidence of healing and resolution of clinical signs. Although *Brucella canis* is not a rare disease in dogs, the documentation of two out of three adult littermates with associated discospondylitis is an interesting feature. In addition, this report highlights available diagnostic and treatment options, as each patient was managed differently based on clinical signs and the preference of the managing clinician.

## Introduction

Discospondylitis is a primary infection of the vertebral endplates with secondary extension into the intervertebral disc space (1–3). A majority of cases develop when an infectious organism spreads hematogenously to a vertebral endplate; however, in rare instances, the infection may start within the intervertebral disc and spread to the adjacent endplates (1). The most common bacterial species isolated is *Staphylococcus*; however, *Streptococcus, E. coli*, and *Brucella canis* have been identified as causative agents, among others (1–3). In any patient diagnosed with discospondylitis, blood and urine cultures are recommended; however, combined blood and urine culture yield a variable success rate in isolating the causative agent, ranging from 30 to 78% (2). Fluoroscopically guided percutaneous intervertebral disc aspiration has shown to be a valuable method of obtaining culture samples in patients with discospondylitis, especially in dogs with negative blood and urine cultures (4). Although known to be a cause of discospondylitis, *Brucella canis* is an uncommon bacterial isolate. In a retrospective study evaluating 135 dogs with discospondylitis, *Brucella canis* was deemed to be the causative agent in 14 dogs (5); although due to the difficulty in isolating the organism, this may have been an underestimation (6, 7). Serologic testing consisting of rapid slide agglutination test (RSAT), 2-mercaptoethanol rapid slide agglutination test (2ME-RSAT), and cytoplasmic agar gel immunodiffusion (AGID-cp) is a commonly utilized diagnostic method. RSAT is considered the most sensitive serologic test but may yield false positives (6). When RSAT is positive, a more specific 2ME-RSAT is recommended. AGID is the preferred confirmatory test when RSAT and 2ME-RSAT are positive (5–7). Tube agglutination testing (TAT), microagglutination testing (MAT), indirect fluorescent antibody (IFA) testing, and enzyme-linked immunosorbent assay (ELISA) are additional serologic tests that have also been performed for the diagnosis of *Brucella canis* (7).

To our knowledge, this is the first report of *Brucella canis* being isolated from multiple adult littermates, two of which were diagnosed with discospondylitis. While there is another case report documenting *Brucella canis* in two littermate puppies, both puppies were subclinical (8). Outbreaks of *Brucella canis* have also been documented within breeding kennels in various countries, though affected littermates were not specifically identified (9–11). This case report describes the diagnostic and treatment methods used in three adult littermates with *Brucella canis*, and the challenges to eliminate the infection. Out of the three littermates, two were diagnosed with discospondylitis, which serves as a reminder to consider *Brucella canis* in any dog with discospondylitis, regardless of age or neuter status.

## Case descriptions, diagnostic interpretation, and therapeutic intervention

### Patient #1

Patient #1, a 2-year-old spayed female Labrador Retriever, presented to the University of Illinois Veterinary Teaching Hospital for episodes of cervical pain and lethargy, which were first noted at 7 months of age. The episodes progressed to include circling to the right and a right-sided head tilt. Each episode lasted a few days in duration followed by complete resolution. Clinical signs were initially managed with carprofen (Rimadyl^®^ Zoetis, Kalamazoo, MI) at 1.6 mg/kg PO q12h and methocarbamol (Robaxin^®^ Zoetis, Kalamazoo, MI) at 16 mg/kg PO q12h. Approximately 3 months before the initial neurologic evaluation, the patient experienced another episode, during which a brief period of presumptive nystagmus was noted. Due to concerns of increased discomfort, gabapentin (10 mg/kg PO q8h) was prescribed. Video evidence of the nystagmus and circling episodes was unavailable. Due to the increase in severity and duration of clinical signs, the patient was referred for further evaluation. Upon presentation, the patient's physical and neurologic examinations were unremarkable. To yield the most useful diagnostic information, all medications were discontinued and the clients were instructed to return during the next episode for further evaluation and neurodiagnostic workup. The patient represented 6 weeks later as the signs returned and had been present for 10 days in duration. At this time, the neurologic exam revealed a slight right-sided head tilt (estimated 10–15 degrees), neck guarding with muscle fasciculations, and decreased range of motion in the dorsal direction. The remainder of the neurologic exam was unremarkable. Positional nystagmus was not appreciated during either of the examinations. Based on the examination findings, a problem affecting the cervical spine was considered. This could include pathology of the bones and/or soft tissues surrounding the cervical spine, pain from meningitis, or compressive spinal cord disease. While the patient did not demonstrate changes to support myelopathy, one consideration for the reported episode of nystagmus was pathology along the medial vestibulospinal tract within the parenchyma of the cervical spinal cord. An alternative consideration was an additional lesion in the central or peripheral vestibular system, though no other vestibular signs other than the head tilt, which can manifest in patients with cervical discomfort or vestibular dysfunction, were observed.

**Figure 1:**
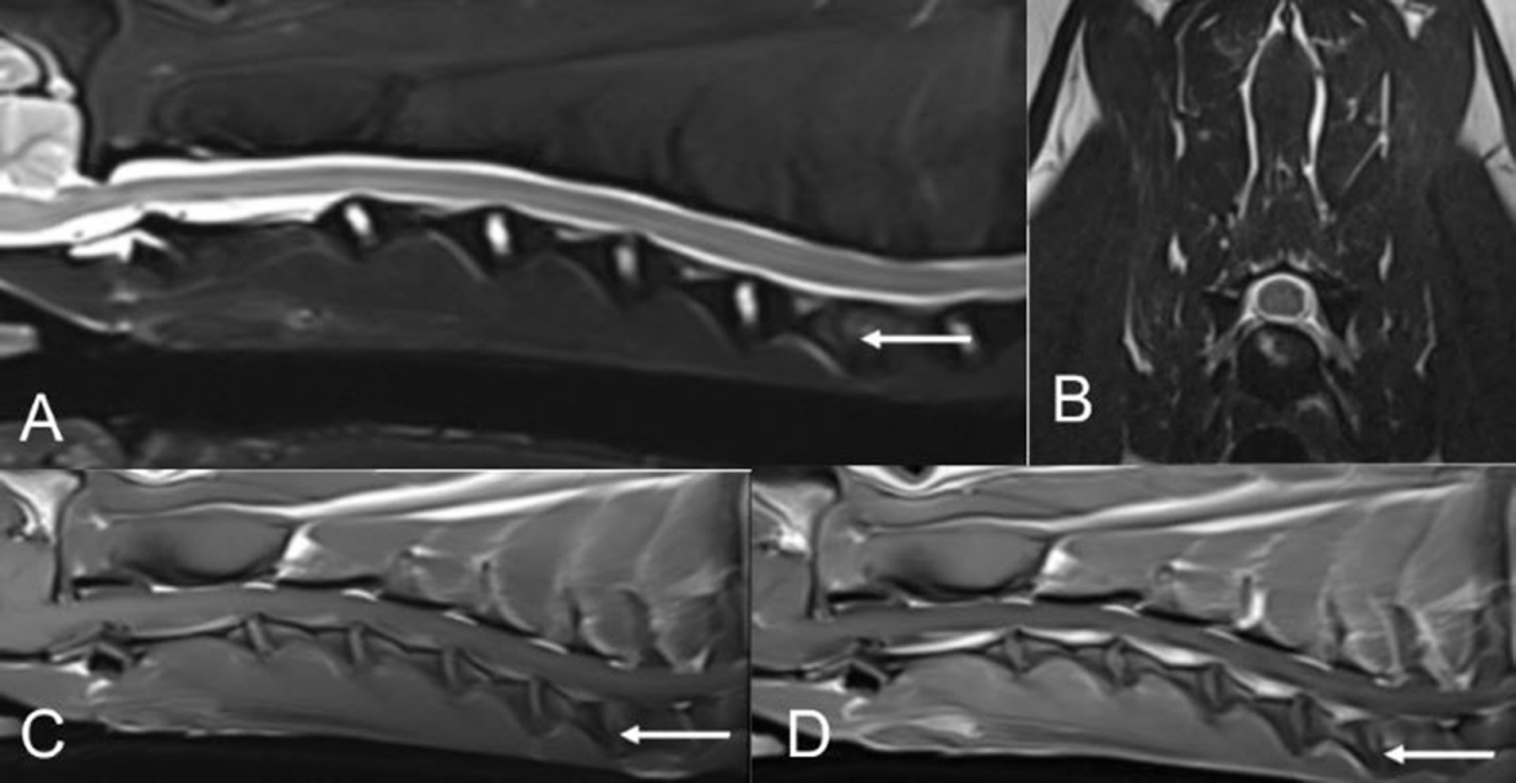
MR images of the cervical spine from patient #1. **(A,B)** T2W sagittal and T2W transverse (at the level of the C6-C7 intervertebral disc space). **(C)** T1W sagittal pre-contrast. **(D)** T1W sagittal post-contrast.

Based on this patient's examination findings and history, magnetic resonance imaging (MRI) of the brain and cervical spine was recommended. Before MRI, a venous blood gas, packed cell volume, and total solids were performed and were unremarkable. MRI of the cervical spine and brain was performed using a 3.0 Tesla magnet (MAGNETOM^®^ Skyra, Siemens. Munich, Germany). Results of the cervical MRI revealed T2W hyperintensity, T1W mixed hypo-iso-intensity, and T1W post-contrast enhancement of the endplates of the caudal C6 and cranial C7 vertebral bodies with irregularity in the structure of the dorsal aspects of the endplates and the corresponding intervertebral disc (Figure 1). These findings were consistent with discospondylitis of the C6-C7 intervertebral disc space and associated vertebral bodies. The brain appeared unremarkable. A cause for the patient's head tilt, reported circling, and nystagmus was not determined based on these findings. Cerebrospinal fluid (CSF) analysis would have been beneficial and was recommended to evaluate for meningoencephalomyelitis, but the patient's family declined.

A blood sample was aseptically collected and submitted for aerobic, anaerobic, and *Brucella* spp. bacterial cultures. A urine sample was obtained *via* cystocentesis for culture. Pending culture and susceptibility results, generic cephalexin (Aurobindo Pharma, East Windsor, NJ) was prescribed (25 mg/kg PO q12h). Shortly after beginning treatment, the patient became increasingly painful, prompting the addition of enrofloxacin (Baytril^®^ Elanco, Shawnee, KS) at 4.5 mg/kg PO q12h to increase the spectrum of coverage. The patient's clinical signs improved 3 days following this addition.

Aerobic and *Brucella* spp. blood cultures yielded *Brucella* spp. growth. A specific Brucella blood culture, consisting of plating on Brucella agar with 5% sheep blood, in addition to routine blood culture was performed. Polymerase chain reaction (PCR) of the isolate and indirect fluorescent antibody (IFA) testing for *Brucella canis* was used to identify the species. All tests were performed at the Veterinary Diagnostic Lab at the University of Illinois College of Veterinary Medicine and were performed by one of the authors (CM). Due to zoonotic potential and state reporting requirements, the state veterinarian was contacted. Following the diagnosis of *Brucella canis*, cephalexin was discontinued and doxycycline (5 mg/kg PO q12h) was administered concurrently with enrofloxacin, pending results of a susceptibility panel. The results of the panel showed that the *Brucella canis* organism was susceptible to marbofloxacin and had intermediate sensitivity to enrofloxacin; therefore, enrofloxacin was discontinued and marbofloxacin (Zeniquin^®^ Zoetis, Kalamazoo, MI) was prescribed (3.3 mg/kg PO q24h). The panel also showed susceptibility to doxycycline. Based on the favorable response to treatment and susceptibility results, this patient was treated with doxycycline and marbofloxacin for 12 months, and whole blood cultures obtained 6, 12, and 18 months following diagnosis were negative. The patient remained subclinical until ~26 months following diagnosis, at which time she exhibited similar signs as before diagnosis. IFA for *Brucella canis* was submitted and was positive. Doxycycline and marbofloxacin were restarted, and the patient was lost to follow-up thereafter.

### Patient #2

Following the diagnosis of patient #1, the family of patient #2 (who were acquainted with the family of patient #1) was notified and instructed to have patient #2 tested for *Brucella canis*. Patient #2 was not examined by the authors, though was examined by two other services at the same institution within 1 year of diagnosis. Patient #2 had a previous 10-month history of intermittent lumbar and coxofemoral joint pain. Radiographs of the lumbar spine were performed at the onset of signs and were unremarkable. Orthopedic evaluation attributed the joint pain to bilateral hip dysplasia, and no overt neurologic abnormalities were appreciated. In addition to intermittent lumbar and joint pain, this patient developed exophthalmia and a fever 6 months before being diagnosed with *Brucella canis*. A computed tomography (CT) of the head and culture of fluid from the retrobulbar space were consistent with an abscess. *Enterococcus* spp., *Pasturella canis*, and *Actinomyces* spp. were isolated and the abscess resolved following treatment with broad-spectrum antibiotics. After notification of patient #1's diagnosis, blood from patient #2 was submitted for aerobic and *Brucella spp*. cultures, PCR, and IFA. All submitted tests were positive for *Brucella canis* and the state veterinarian was notified. Anaerobic testing was not submitted due to the low clinical suspicion. Patient #2 was asymptomatic at the time of diagnosis. Susceptibility results were similar to those of patient #1; therefore, a 6-month course of marbofloxacin and doxycycline was prescribed. The patient remained PCR positive following 4 months of treatment and repeat cultures were planned following a total of 6 months of treatment. This patient was lost to follow-up before the culture recheck.

### Patient #3

A third littermate (patient #3) was identified with the assistance of patient #1's family. Similar to patient #1, patient #3 was diagnosed with *Brucella canis*-associated discospondylitis. Patient #3 was not examined by the authors, though was evaluated and diagnosed at the institution of one of the authors (KA), making records and imaging results accessible. Patient #3 presented to the University of Tennessee College of Veterinary Medicine at ~18 months of age for a 6-month history of progressive lower back pain when squatting or jumping. Initial neurologic examination revealed hyperesthesia upon lumbosacral palpation and was otherwise unremarkable. In the absence of any other neurologic deficits, a precise neurolocalization was not possible, and a disorder of the lumbosacral region was suspected. Similar to patient #1, an alternative consideration was pathology in the bones, joints, or soft tissues surrounding the lumbosacral spine. Differential diagnoses for this patient included inflammatory disease, congenital malformation, unwitnessed traumatic injury, and less likely neoplasia.

**Figure 2:**
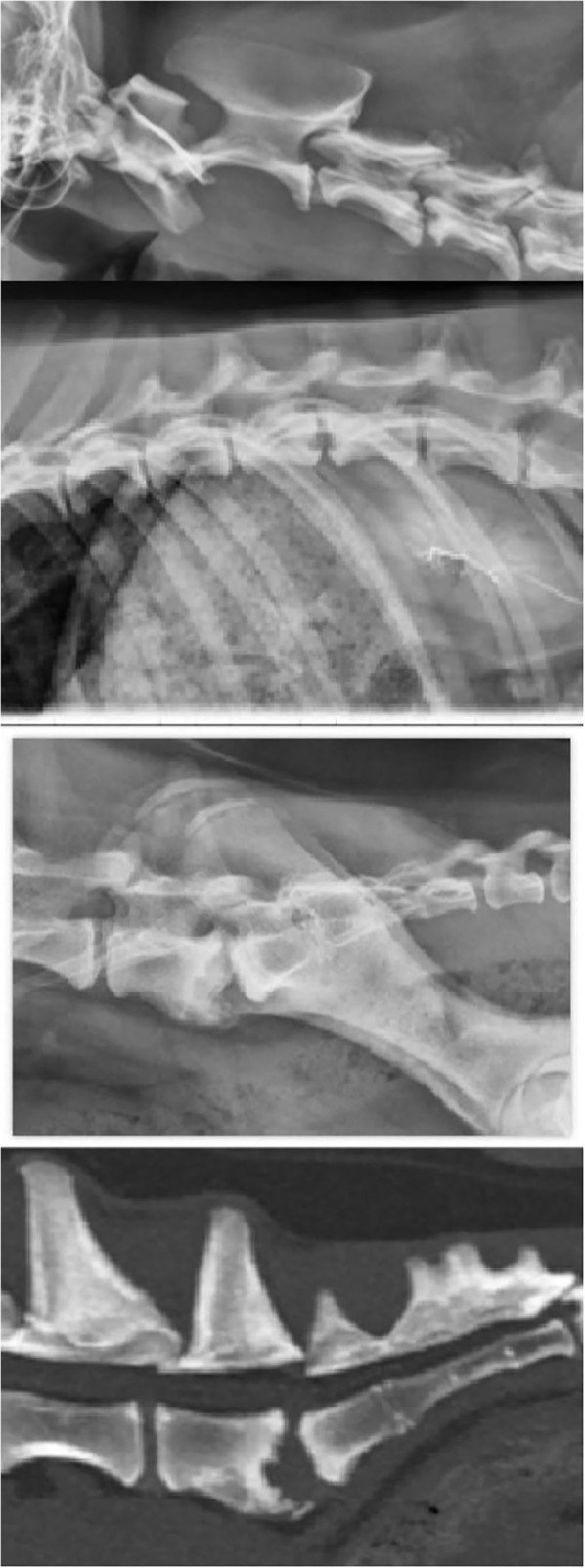
Radiographs from of the cervical, thoracolumbar, and lumbosacral spine, and CT of the lumbosacral spine (top to bottom) from patient #3.

Following patient #3's examination, radiographs of the cervical, lumbar, and thoracic spine were performed and revealed lysis of the vertebral endplates of C3-C4, T12-T13, and L7-S1, consistent with discospondylitis (Figure 2). A CT of the lumbosacral spine was performed. The reason for performing CT only of the lumbosacral spine was unclear upon review of available medical records. The CT scan showed lysis and irregularity within the vertebral endplates at L7-S1, further supporting the diagnosis of discospondylitis (Figure 2). The patient was initially prescribed amoxicillin/clavulanic acid (Clavamox^®^ Zoetis, Parsippany, NJ) at 23 mg/kg PO q12h. Aerobic and anaerobic whole blood and urine (collected *via* cystocentesis) cultures were submitted in addition to *Brucella canis* serologic testing consisting of RSAT, 2ME-RSAT, and AGID-cp. While no growth was present on blood and urine cultures, all submitted serologic tests were positive for *Brucella canis*. Due to the positive serologic results and low clinical suspicion, fungal testing was not performed. Amoxicillin/clavulanic acid was discontinued in favor of enrofloxacin (5.5 mg/kg PO q24h). Spinal radiographs obtained 3 and 6 months following diagnosis showed evidence of osteoproliferation of the endplates at C3-C4 and L7-S1, while the lesion at T12-T13 remained static. In addition, no new lesions were observed and the patient was reportedly free of clinical signs. Enrofloxacin was administered at the initial dosage for a total of 7 months and was discontinued thereafter. At the time of discontinuation, the patient was asymptomatic and was lost to follow-up thereafter.

## Discussion

In this case report, we presented three adult canine littermates who tested positive for *Brucella canis*, two of which were diagnosed with discospondylitis. While a variety of diagnostic tests have been utilized for the detection of *Brucella canis*, it is often difficult to diagnose due to the low and variable number of infected leukocytes in circulation and the organism's tendency to reside in tissues (6, 7). Definitive diagnosis relies on isolating the organism from whole blood or other infected tissues and fluids; however, *Brucella canis* tends to grow slowly and is fastidious for culture (6, 7). In patients #1 and #2, blood cultures were not positive until 5 days of incubation. Based on author (a PhD microbiologist, CM) experience and available literature, Brucella agar and incubation in a 5% CO_2_ environment may also be required to encourage growth (12). Due to these diagnostic challenges, a single negative culture may not rule out *Brucella canis* (6, 7, 13) and some authors recommend submitting a series of three blood cultures collected at least 24 h apart before a negative result is confirmed (6). Serology and PCR are additional diagnostic options (7). Some of the most commonly utilized serologic tests include MAT, RSAT, 2-ME RSAT, and AGID. Sensitivity and specificity for MAT, respectively, are 66.7–88.9% and 100% (14, 15). The reported sensitivity and specificity for RSAT are 70.6% and 83.34%. For 2-ME RSAT and AGID, the sensitivity and specificity are 31.8% and 100%, and 52.9% and 100%, respectively (16). IFA is typically used as a screening test due to its high sensitivity, making RSAT the reference test to confirm or rule out positive IFA samples. ELISA has been utilized and sensitivity and specificity vary based on the antigen used (17, 18). While specific PCRs for *Brucella canis* have been developed, the sensitivity and specificity of PCR for *Brucella canis* have not been determined in canine populations (17, 19–21). Furthermore, PCR results may depend on whether patients are bacteremic at the time of sampling, have been treated with recent antibiotics, and whether PCR inhibitors are present in the sample (14, 18). Therefore, serology may be preferred as it can provide more rapid results and is more widely available over PCR.

Discospondylitis generally requires up to a year of antimicrobial treatment, with a reported mean duration of 53.7 ± 45.4 weeks (2) regardless of the etiologic agent. *Brucella canis-*associated discospondylitis tends to be even more challenging to treat as the organism may be harbored in tissues for extended periods of time and despite long-term treatment, and the patient may never clear the infection even after the resolution of clinical signs (5) and seronegative conversion (7). The organism's tendency to recrudesce was exemplified in patient #1 when signs returned over 2 years following initial diagnosis despite chronic treatment with doxycycline and marbofloxacin. *Brucella canis-*associated discospondylitis often requires multiple classes of antibiotics, and historically a combination of tetracyclines and aminoglycosides has been effective in the resolution of clinical signs (1, 5). Combination therapy with doxycycline and rifampicin combined with surgical resection of infected tissues is effective in treating *Brucella suis* in dogs (22). Current World Health Organization recommendations for human brucellosis are combination therapy with either doxycycline and rifampicin or doxycycline and streptomycin (23). As an alternative to combination therapy in dogs, monotherapy with enrofloxacin has demonstrated similar efficacy for *Brucella canis*, preserved fertility, and prevented bacterial dissemination in a group of dogs following a kennel outbreak (9). Based on susceptibility results, marbofloxacin was used in place of enrofloxacin, in patients #1 and #2, who were also treated with doxycycline. Patient #3 was treated with enrofloxacin alone. While treatment with fluoroquinolones has shown some success in managing clinical signs in dogs, judicious use is imperative due to the growing resistance to fluoroquinolones (24, 25). It should be noted that fluoroquinolones were unable to achieve long-term control in our patients as one patient remained bacteremic during treatment and another patient relapsed a year after discontinuation of treatment.

The patients presented in this case report varied from the common presentation of discospondylitis from other causes, being female, and <2 years of age at the time of diagnosis. Males are at least two times as likely as females to be diagnosed with discospondylitis (2, 5), and most patients are middle-aged or older, with a mean age of 6.8 years reported in one study (26) and an odd's ratio highest in dogs over 10 years in another (2). The two patients with discospondylitis in this report were noted to have lesions in the cervical spine (C6-C7 for patient #1 and C3-C4 for patient #3), which is typically the least common section of the spinal cord to be affected. Patient #3 also had lesions in the more commonly affected thoracic and lumbosacral regions. Thoracolumbar imaging was not performed in patient #1, therefore undiagnosed lesions may have been possible. In a retrospective study of 513 dogs diagnosed with discospondylitis, a majority of lesions were located in the thoracic and lumbar vertebral bodies with the L7-S1 segment being the most commonly affected site. Cervical vertebrae were affected in only 13.8% of the dogs in this study (2). In another study, lesions of the thoracic and lumbar spine were also more commonly reported than those in the cervical spine (26). Interestingly, a paper by Buhmann et al. also reported brucella-associated discospondylitis in 4 young females, ranging from 7 months to 2.5 years of age, and 3 of the 4 had lesions in the cervical spine (27).

We have documented three adult littermates who were diagnosed with *Brucella canis*, two of which developed discospondylitis. Despite its diagnostic challenges, *Brucella canis* was identified in all three littermates using various diagnostic methods (including blood culture, PCR, and multiple serologic tests). When dogs are diagnosed with *Brucella canis*, treatment can be challenging and requires chronic, and potentially life-long, antibiotic administration without guarantee of clearance of the organism. The need for a reliable and consistent diagnostic protocol for *Brucella canis* remains, and could prove beneficial for veterinarians should the need for testing one or multiple patients arise.

## Data availability statement

The original contributions presented in the study are included in the article/supplementary material, further inquiries can be directed to the corresponding author/s.

## Author contributions

LG was responsible for drafting the manuscript. All authors read and approved the final version of the manuscript.

## Conflict of interest

The authors declare that the research was conducted in the absence of any commercial or financial relationships that could be construed as a potential conflict of interest.

## Publisher's note

All claims expressed in this article are solely those of the authors and do not necessarily represent those of their affiliated organizations, or those of the publisher, the editors and the reviewers. Any product that may be evaluated in this article, or claim that may be made by its manufacturer, is not guaranteed or endorsed by the publisher.

## References

[1] Thomas W. Diskospondylitis and other vertebral infections. Vet Clin North Am Small Anim Pract. (2000) 30:169–82. 10.1016/S0195-5616(00)50008-4 10680214

[2] Burkert BA Kerwin SC Hosgood GL Pechman RD Fontenelle JP. Signalment and clinical features of diskospondylitis in dogs: 513 cases (1980-2001). J Am Vet Med Assoc. (2005) 227:268–75. 10.2460/javma.2005.227.268 16047665

[3] Ruoff CM Kerwin SC Taylor AR. Diagnostic imaging of discospondylitis. Vet Clin North Am Small Anim Pract. (2018) 48:85–94. 10.1016/j.cvsm.2017.08.007 28964545

[4] Fischer A Mahaffey MB Oliver JE. Fluoroscopically guided percutaneous disk aspiration in 10 dogs with diskospondylitis. J Vet Intern Med. (1997) 11:284–7. 10.1111/j.1939-1676.1997.tb00466.x 9348495

[5] Kerwin SC Lewis DD Hribernik TN Partington B Hosgood GL Eilts BE. Diskospondylitis associated with *Brucella canis* infection in dogs: 14 cases (1980-1991). J Am Vet Med Assoc. (1992) 201:1253–7. 1429171

[6] Hollett RB. Canine brucellosis: outbreaks and compliance. Theriogenology. (2006) 66:575–87. 10.1016/j.theriogenology.2006.04.011 16716382

[7] Makloski CL. Canine brucellosis management. Vet Clin North Am Small Anim Pract. (2011) 41:1209–19. 10.1016/j.cvsm.2011.08.001 22041212

[8] Dentinger CM Jacob K Lee LV Mendez HA Chotikanatis K McDonough PL. Human *Brucella canis* infection and subsequent laboratory exposures associated with a puppy, New York City, 2012. Zoonoses Public Health. (2015) 62:407–14. 10.1111/zph.12163 25363807 PMC4639931

[9] Wanke MM Delpino MV Baldi PC. Use of enrofloxacin in the treatment of canine brucellosis in a dog kennel (clinical trial). Theriogenology. (2006) 66:1573–8. 10.1016/j.theriogenology.2006.01.034 16476476

[10] Weese JS Hrinivich K Anderson MEC. *Brucella canis* in commercial dog breeding kennels, Ontario, Canada. Emerg Infect Dis. (2020) 26:3079–80. 10.3201/eid2612.201144 33219799 PMC7706960

[11] Keid LB Chiebao DP Batinga MCA Faita T Diniz JA Oliveira TMF de S. Brucella canis infection in dogs from commercial breeding kennels in Brazil. Transbound Emerg Dis. (2017) 64:691–7. 10.1111/tbed.12632 28296215

[12] Theron J Cloete T. Brucella characteristics. In: Encyclopedia of Food Microbiology. Cambridge, MA: Academic Press (1999). p. 319–24. 10.1006/rwfm.1999.0255

[13] Wanke MM. Canine brucellosis. Anim Reprod Sci. (2004) 82–83:195–207. 10.1016/j.anireprosci.2004.05.005 15271453

[14] Mol JPS Guedes ACB Eckstein C Quintal APN Souza TD Mathias LA. Diagnosis of canine brucellosis: comparison of various serologic tests and PCR. J Vet Diagn Invest. (2020) 32:77–86. 10.1177/1040638719891083 31752635 PMC7003229

[15] Castillo Y Tachibana M Kimura Y Kim S Ichikawa Y Endo Y. Microplate agglutination test for canine brucellosis using recombinant antigen-coated beads. Int Scholarly Res Notices. (2014) 2014:1–4. 10.1155/2014/348529 27355048 PMC4897435

[16] Keid LB Soares RM Vasconcellos SA Megid J Salgado VR Richtzenhain LJ. Comparison of agar gel immunodiffusion test, rapid slide agglutination test, microbiological culture and PCR for the diagnosis of canine brucellosis. Res Vet Sci. (2009) 86:22–6. 10.1016/j.rvsc.2008.05.012 18656213

[17] Cosford KL. Brucella canis: An update on research and clinical management. Can Vet J. (2018) 59:74–81. 29302106 PMC5731389

[18] Santos RL Souza TD Mol JPS Eckstein C Paíxão TA. Canine brucellosis: an update. Front Vet Sci. (2021) 8:594291. 10.3389/fvets.2021.594291 33738302 PMC7962550

[19] Kaden R Ågren J Båverud V Hallgren G Ferrari S Börjesson J. Brucellosis outbreak in a Swedish kennel in 2013: determination of genetic markers for source tracing. Vet Microbiol. (2014) 174:523–30. 10.1016/j.vetmic.2014.10.015 25465667

[20] Kang SI Lee SE Kim JY Lee K Kim JW Lee HK. A new Brucella canis species-specific PCR assay for the diagnosis of canine brucellosis. Compar Immunol Microbiol Infect Dis. (2014) 37:237–41. 10.1016/j.cimid.2014.07.003 25128932

[21] Kauffman LK Bjork JK Gallup JM Boggiatto PM Bellaire BH Petersen CA. Early detection of brucella canis via quantitative polymerase chain reaction analysis. Zoonoses Public Health. (2014) 61:48–54. 10.1111/zph.12041 23409865

[22] James D Golovsky G Thornton J Goodchild L Havlicek M Martin P. Clinical management of Brucella suis infection in dogs and implications for public health. Aust Vet J. (2017) 95:19–25. 10.1111/avj.12550 28124423

[23] Brucellosis. World Health Organization (2020). Available online at: https://www.who.int/news-room/fact-sheets/detail/brucellosis (accessed August 6, 2022).

[24] Hooper DC Jacoby GA. Topoisomerase inhibitors: fluoroquinolone mechanisms of action and resistance. Cold Spring Harb Perspect Med. (2016) 6:1–21. 10.1101/cshperspect.a025320 27449972 PMC5008060

[25] Redgrave LS Sutton SB Webber MA Piddock LJV. Fluoroquinolone resistance: mechanisms, impact on bacteria, and role in evolutionary success. Trends Microbiol. (2014) 22:438–45. 10.1016/j.tim.2014.04.007 24842194

[26] Harris JM Chen AV Tucker RL Mattoon JS. Clinical features and magnetic resonance imaging characteristics of diskospondylitis in dogs: 23 cases (1997–2010). J Am Vet Med Assoc. (2013) 242:359–65. 10.2460/javma.242.3.359 23327179

[27] Buhmann G Paul F Herbst W Melzer F Wolf G Hartmann K. Canine brucellosis: insights into the epidemiologic situation in Europe. Front Vet Sci. (2019) 6:151. 10.3389/fvets.2019.00151 31214601 PMC6554662

